# STEMI or non-STEMI: that is the question

**DOI:** 10.1007/s12471-015-0665-x

**Published:** 2015-03-04

**Authors:** Cyril Camaro, Menko-Jan de Boer

**Affiliations:** Department of Cardiology, Section Interventional Cardiology, Radboud University Medical Center, PO Box 9101, 6500 HB Nijmegen, The Netherlands

**Keywords:** ST-segment elevation myocardial infarction, Non ST-segment elevation myocardial infarction, Acute coronary syndrome, Primary percutaneous coronary intervention, Risk stratification

## Abstract

Acute coronary syndromes are usually classified on the basis of the presence or absence of ST elevation on the ECG: ST-elevation myocardial infarction or non-ST-elevation myocardial infarction (NSTEMI)patients with acute myocardial infarction (AMI) need immediate therapy, without unnecessary delay and primary percutaneous coronary intervention (PPCI) should preferably be performed within 90 min after first medical contact. However, in AMI patients without ST-segment elevation (pre) hospital triage for immediate transfer to the catheterisation laboratory may be difficult. Moreover, initial diagnosis and risk stratification take place at busy emergency departments and chest pain units with additional risk of ‘PPCI delay’. Optimal timing of angiography and revascularisation remains a challenge. We describe a patient with NSTEMI who was scheduled for early coronary angiography within 24 h but retrospectively should have been sent to the cath lab immediately because he had a significant amount of myocardium at risk, undetected by non-invasive parameters.

## Case

**Figure 1:**
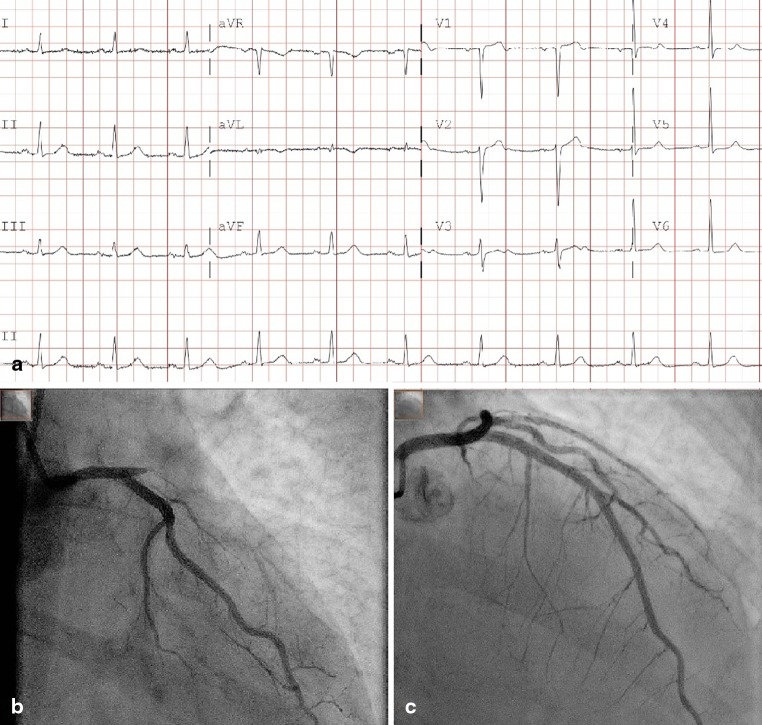
a Electrocardiogram on admission. 25 mm/s, 10 mm/mV. b Left coronary artery in RAO caudal angulation. Before intervention. c Left anterior descending artery in RAO cranial view. After PCI with implantation of a 3.5 mm drug-eluting stent

A 70-year-old male was diagnosed with non-ST-elevation myocardial infarction (NSTEMI) without signs of haemodynamic compromise and intermediate GRACE risk scores (Fig. 1a). Initial cardiac biomarkers were elevated with a creatine kinase of 1236 U/l and positive high sensitive troponin of 787 ng/l. He was scheduled for coronary angiography within 24 h. One and a half hours after admission the pain had not resolved despite medical therapy, and it was decided to perform immediate angiography. To our surprise, occlusion of a large left anterior descending artery (LAD) was found with collaterals from the right coronary artery. Subsequent successful percutaneous coronary intervention of the LAD was performed (Fig. 1b and c). The procedure was successful with TIMI-3 flow and myocardial blush grade 3. After the procedure the patient remained free of symptoms and during further observation no complications occurred.

## Conclusion

ST-segment elevation only may not always reflect ongoing ischaemia and we should no longer focus on the presence or absence of ST-segment elevation as a reliable criteria to proceed or to postpone urgent angiography and/or reperfusion therapy [1, 2]. Future studies should focus on the NSTEMI ACS algorithm and its identification of high-risk patients who may benefit from urgent coronary angiography and subsequent revascularisation [3, 4, 5]. In our opinion, the acute myocardial infarction classification based on ST elevation alone should be reconsidered.
